# Life-stage and environmental influences on the recruitment of African freshwater eels into the uThukela River, South Africa

**DOI:** 10.1111/jfb.70239

**Published:** 2025-09-30

**Authors:** Rory McNeill, Matthew J. Burnett, Céline Hanzen, Colleen T. Downs

**Affiliations:** 1 Centre for Functional Biodiversity, School of Life Sciences, University of KwaZulu-Natal, Pietermaritzburg, South Africa; 2 Ecosystems, Institute of Natural Resources NPC, Scottsville, South Africa

**Keywords:** anguillid, elvers, glass eels, seasonality

## Abstract

Anguillid eel recruitment into east-flowing rivers along the east coast of Africa is poorly understood. The few harvest records of anguillid eels from South Africa have highlighted anthropogenically derived environmental stressors as risks for further decline. Our study aimed to determine environmental conditions and elver development associated with eel recruitment in the uThukela River, KwaZulu-Natal, South Africa. Our study was conducted between November 2021 and September 2022 and compared the effect of high- and low-flow conditions and the associated environmental variables on eel recruitment. We captured 519 eels and identified 220 (42%) which were classified into three species (*Anguilla bengalensis n* = 51, *Anguilla marmorata n* = 60 and *Anguilla mossambica n* = 120). We captured glass eels (*n* = 461) and elvers (*n* = 36), and included transitioning individuals (*n* = 22) between the stages. All eels were caught in high-water-flow conditions, which supports the hypothesis that eels prefer high water flow for recruitment, which usually occurs in rainy, warm austral summer months. We found a significant difference between the body lengths of the three identified species. We found a significant difference between the life stages present, which was primarily between glass eel and elver, with the transitional phase grouping in body length relative to glass eel body length. Our results highlighted the need to improve field identification of glass eels. In addition, an in-depth understanding of the recruitment trends for African freshwater eels in east-flowing rivers is needed.

## 1 Introduction

Anguillid eels have a global distribution, with four species of anguillid freshwater eels occurring along the east coast of Africa in the Western Indian Ocean. These are (1) Mozambique eel (*Anguilla mossambica*, Peters 1852), (2) African Bengal eel (*Anguilla bengalensis*, Peters 1852), (3) Indo-Pacific eel (*Anguilla marmorata*, Quoi Griman 1824) and (4) Indian bicolour eel (*Anguilla bicolor*, McClelland 1844). *Anguilla mossambica* is the only endemic species to the South-West Indian Ocean, whereas the other species have a more extensive range distribution and can be found as far as the Pacific Ocean (Arai, 2020; Hanzen et al., 2021; Lin et al., 2012; Pous et al., 2010). The four African eel species are transported as leptocephalus larvae from their spawning areas and metamorphose into glass eels when they recruit into rivers along the east coast of Africa as far south as Cape Town in South Africa (Hanzen et al., 2019; Lin et al., 2012; Skelton, 2024). Anguillid eels are the only long-distance catadromous species in the Western Indian Ocean (Hanzen et al., 2019). The factors that drive the recruitment of glass eels from the marine environment into estuaries are poorly understood, especially in South Africa, and the need for further research has been identified (Hanzen et al., 2019; Righton et al., 2021; Robinet, Guyet, et al., 2003; Robinet, Lecomte-Finiger, et al., 2003).

Upon arrival in the estuarine environment, glass eels have a weak swimming ability, making it difficult for them to move upstream, especially when there are prolonged periods of strong downstream currents (Harrison et al., 2014; McCleave, 1980; Wuenschel & Able, 2008). To compensate for their weak swimming ability, glass eels use a process known as ‘selective tidal stream transport’, which conserves their energetic requirements (Briand et al., 2005; McCleave & Kleckner, 1982). During this transport process, glass eels move upstream with the incoming tide and take refuge near the bottom or by burrowing into the substrate during the outgoing tide to preserve energy (Beaulaton & Castelnaud, 2005; Deelder, 1958; Harrison et al., 2014). Certain factors, such as increased distance from the estuary into the freshwater environment and high water flow, reduce the selective tidal stream transportation (Beaulaton & Castelnaud, 2005; Gascuel, 1986; McCleave & Kleckner, 1982). At a specific point when selective tidal stream transportation is decreased sufficiently, glass eels aggregate into shoals, swim closer to the shore and begin a more active migration upstream towards fresh water (Beaulaton & Castelnaud, 2005; Verhelst et al., 2018).

As glass eels move upstream, they develop into elvers, which is not always fixed and predictable (Robinet, Guyet, et al., 2003; Robinet, Lecomte-Finiger, et al., 2003). There are an increasing number of studies that provide evidence that eels have a more diverse and unpredictable migratory life cycle than previously thought (Arai, 2020; Arai et al., 2020; Arai & Chino, 2012; Daverat et al., 2006; Feunteun et al., 2003; Jessop et al., 2008; Lin et al., 2012; Marohn et al., 2013; Robinet, Guyet, et al., 2003; Robinet, Lecomte-Finiger, et al., 2003; Tsukamoto et al., 1998; Tzeng et al., 1996; Yokouchi et al., 2012). Penetration upstream into rivers by elvers is variable and species dependent, with some species migrating further upstream than others (Davey & Jellyman, 2005; Hanzen et al., 2022; Harrison et al., 2014; Jellyman, 2021). The factors affecting the distances migrated are presently not quantified, although the recruitment abundance of glass eels into a river is necessary and influences upstream movement.

During the eels marine phase as they move towards freshwater ecosystems their movement is influenced by various factors, including the earth’s magnetic field, the lunar phase, olfactory cues and salinity gradients (Cresci et al., 2017, 2019; Edeline et al., 2009; Sola & Tosi, 1993). Similarly, the recruitment time is influenced by various factors, such as estuary salinity levels, thermal dynamics, estuarine length, spatial complexity, tidal magnitude and freshwater inputs (Harrison et al., 2014). Research into site and species-specific recruitment becomes necessary as results from one study cannot be extrapolated to another. Research is also needed as studies show that recruitment of European glass eels is declining, and an extensive, collaborative report from the Working Group on Eels provided data from a time series from 1980 to 2023 and showed that glass eel recruitment is declining in the North Sea (ICES, 2023). In the Bay of Biscay, a northeastern embayment of the Atlantic Ocean along the coast of France and Spain, there is an indication that glass eel recruitment has increased (Aranburu et al., 2016). However, glass eel recruitment varies interannually (Bouchard et al., 2022). There are a few studies and data on glass eel recruitment in South Africa, nor the rest of mainland Africa (Hanzen et al., 2019).

In South Africa, ~289 estuaries that stretch across a wide latitude range (Reddering & Rust, 1990) are available for glass eels to recruit into. These estuaries have various environmental factors that will affect glass eel recruitment, and in addition, the estuaries face numerous anthropogenic threats, including pollution, habitat loss and fishing (de Lecea & Cooper, 2016; Lamberth et al., 2009; Vezi et al., 2020; Wade et al., 2021). Anthropogenic threats (e.g. pressure to meet increasing industrial, mining, agricultural and domestic water demands) have resulted in heavily modified rivers in South Africa and reduced ecological functionality (Evans et al., 2022; Van Deventer et al., 2018). The estuaries in South Africa are generally poorly conserved, and estuarine modification can impede the breeding or recruitment of many species, including eels (Bate et al., 2011). Consequently, anthropogenic threats pose potential risks to eel’ recruitment into South African estuaries (Hanzen et al., 2022). One important river in South Africa for all four species of African eels is the uThukela Catchment, as it is one of the few relatively free-flowing rivers remaining in South Africa (de Lecea & Cooper, 2016; Evans et al., 2022; Nel et al., 2011). It is also one of the few catchments where the four species co-occur (Hanzen et al., 2022). A recent development that poses a risk to the recruitment of African eels is the construction of a weir for a water supply scheme upstream of the uThukela Estuary. Although the weir is equipped with a rock ramp specifically to accommodate the migratory needs of aquatic fauna requiring a wetted surface to move over instream barriers, including African eels, efficacy concerns remain (Burnett et al., 2024). Unfortunately, the uThukela Estuary ecological health is increasingly being degraded by multiple stressors associated with industry effluent, changes in land use, poor wastewater treatment and altered flows, leading to a cause for concern (de Lecea & Cooper, 2016; Lamberth et al., 2009; Stryftombolas, 2010; Vezi et al., 2020; Wade et al., 2021). There is concern about the present status of African eels in the uThukela Catchment, with some evidence showing that they are declining (Hanzen et al., 2022).

Our study focused on the important recruitment stage of glass eels, a critical and vulnerable stage in their life cycle, into the uThukela Estuary, South Africa, to improve our understanding of the ecology of freshwater eels locally and in the Western Indian Ocean region. We aimed to characterise the timing and prevalence of the recruitment of anguillid eels into the uThukela Estuary and determine possible threats to their recruitment. In addition, we address specific topics relating to glass eels developing into elvers and conditions associated with this, for example, water temperature, light quality and water flow, that could affect recruitment. We predicted that eel recruitment would be influenced by factors associated with water temperature, light quality and water flow.

## 2 Methods

### 2.1 Study area

**Figure 1:**
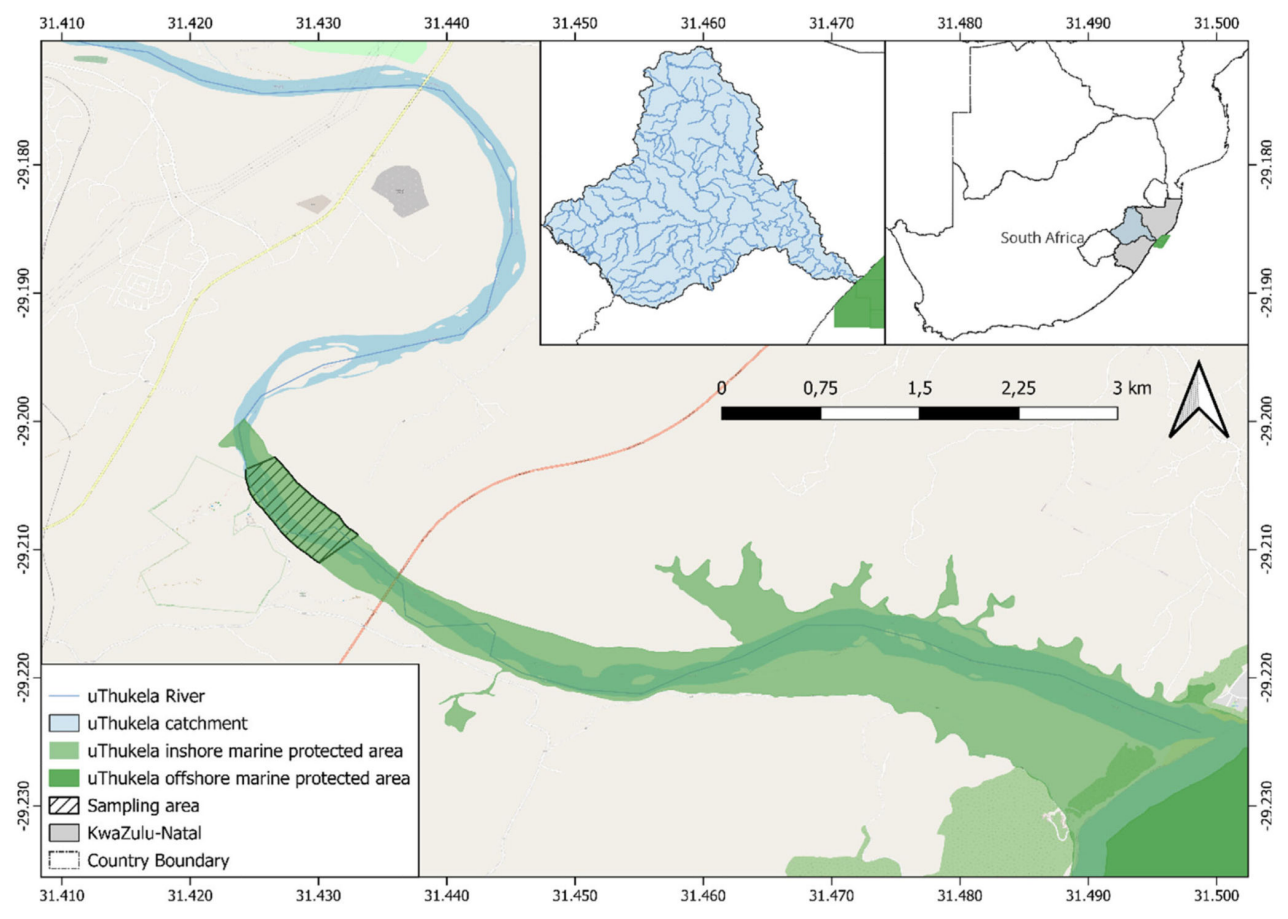
A map showing the uThukela River estuary and study site in the KwaZulu-Natal Province, South Africa.

The uThukela River is the largest river in KwaZulu-Natal Province, along the east coast of South Africa (de Lecea & Cooper, 2016; Figure 1). The river importantly provides nutrients in the form of organic matter that is essential to marine life and offshore fisheries along the coastline (de Lecea & Cooper, 2016; Lamberth et al., 2009; Wade et al., 2021). It has a few impoundments/dams from source to sea, making it relatively free of considerable instream barriers (de Lecea & Cooper, 2016; Nel et al., 2011). All four eel species have historically been found in the river system in relatively high abundance (Hanzen et al., 2022) and are in the southwestern distribution of the global range. Our study site was the uThukela River, which is important for eels: a report by Hanzen et al. (2019) collated 533 eel records from 1957 to June 2020 in KwaZulu-Natal and noted that the uThukela River was important in having 28.9% of all records (*n* = 154). Despite this, the river is under continual threat from anthropogenic stressors, for example, poor water quality from a paper mill, flow alterations from abstraction for industry and water security, sand mining impacting habitat, fishing and invasion by alien fishes (de Lecea & Cooper, 2016; Stryftombolas, 2010; Wade et al., 2021). In addition to these threats, the river is sub-tropically based and experiences extreme climatic events, such as severe drought as recently as 2016 and excessive flooding as recently as the present study in 2022 (Ndlovu & Demlie, 2020; Thoithi et al., 2023).

Our study was undertaken ~8 km upstream of the tidal zone in the river mouth of the uThukela Estuary (Figure 1). This is characterised by a 1:7000 slope with sand bars and slow-flowing water during the low-flow season (Bagnold, 1980). The upper section of the uThukela River is characterised by a 1:10 slope, with a 1:1000 slope in the mid-sections. The river starts at an altitude >3000 m and is 560 km long; it drops 1700 m in the last 480 km and has a catchment of 28,000 km^2^ (DWAF, 2002). The catchment has a mean annual precipitation ranging from 1500 mm in the west to 750 mm in the east. The tidal influence depends on the river inflows to the estuary; however, the estuarine limit is ~9 km from the shoreline (CoastKZN, 2024). This location lies in the uThukela Inshore Controlled Zone, an inshore portion of the uThukela Marine Protected Area. The uThukela Marine Protected Area was created in 2019 and is ~4000 km^2^. It was created to protect interconnected coastal, shelf and slope ecosystems directly associated with the uThukela River Marine interface (Green et al., 2022).

### 2.2 Sampling techniques

We obtained ethical clearance for this study from the University of KwaZulu-Natal Animal Ethics Committee (AREC/023/020). We conducted four night sampling surveys during 8–11 November, 3–6 December 2021 and 7–8 March 2022 (interrupted by flooding) as high flow and surveys during 15–19 August and 13–17 September 2022 as low flow. No sampling was taken despite our intention to sample from November 2021 to April 2022, as extreme rain and devastating flooding from January through April 2022 made conditions unfavourable for sampling (Thoithi et al., 2023); an attempt was made in March 2022 despite this period being reported as one in which increased eel recruitment occurs in South Africa (Harris & Cyrus, 1995; Wasserman et al., 2011; Wasserman & Strydom, 2011). We then collected data to determine the recruitment of glass eels during the austral winter.

To capture eels, we used eight double-winged fyke glass eel nets. These nets, specifically designed to harvest eels commercially, had a mesh-size of 2 mm, with an opening height of 20 cm and length 23.6 cm, wing length of 4.5 m with an otter guard 5 x 5 cm spacing, the wing and net height of 1.45 m and a three-ringed circular frame fyke basket (1.77 m long, T & L Netmaking, Mooroolbar, Australia). We collected glass eels for five consecutive nights during each sampling period. At the start of each sampling event, in the late afternoon, nets were placed along the riverbank at ~50–100 m intervals, with their openings facing downstream. The wing closest to the bank was extended and tied to the vegetation. The other wing was left open in the current only during the heavy-flow periods; otherwise, it was anchored to a pole inserted into the river’s substrate. A wooden pole separated each wing from the other, keeping the opening clear and unobstructed. Nets were checked and emptied mornings and afternoons for a night and day effort, respectively.

We removed captured eels from the nets and transferred them into a holding container with fresh river water for examination and quantification. To ensure the eels were handled with care, we anaesthetised them using 2-phenoxyethanol (Hanzen et al., 2022). Measurements recorded from recruiting eels were length (mm), species when possible and life stage. The measured lengths form a baseline recording of eels recruiting into the river. A longer length of glass eels may suggest they are ready to migrate upstream and begin the transition into elvers. To identify species, we followed the key developed by Réveillac et al. (2009), which is based on tail pigmentation patterns as glass eels morph into elvers (Elie et al., 1982; Figure S1). Life stages were based on body pigmentation, as it gradually changes during the transition from glass eels into elvers. This gradual change has made classifying and differentiating different life stages difficult. Typically, most research around glass eels and their transition into elvers follows a key developed by Elie et al. (1982) for the European eel, *A. Anguilla*, and was used in the present study. This key separates the transition from glass eel into elver into eight different stages based on the pigmentation pattern observed in the individual. We used pigmentation changes to categorise the eel life cycle stages and followed a key developed by Elie et al. (1982). This key separates the glass eels, elvers and transitioning eels for the European eel. For record purposes, each eel captured was photographed alongside a ruler (mm) to quantify length, and the tail pigmentation was assessed on the live specimen according to Réveillac et al. (2009) key (Figure S1).

Environmental variables were recorded during sampling, including pH, electrical conductivity, total dissolved solids, water temperature, photoperiod and moon phase. The water temperature and photoperiod (the number of daylight hours on the day of sampling) indicate seasonality, whereas the moon phase, expressed as a percentage of illumination as recorded on moon charts, provides insight into the influence of lunar phases. For each sampling effort, we measured and recorded environmental parameters using a hand-held multimeter (HI98129, Hannah Instruments, Woonsocket, USA), which measures pH, electrical conductivity, total dissolved solids and water temperature. We assessed the photoperiod by recording the daylight hours on the study day. We examined moon charts to determine the moon phase on the study day.

### 2.3 Data analyses

Firstly, we employed descriptive analyses to determine each variable’s distribution, means and standard deviations (SD). The normality of distribution was tested using the Shapiro–Wilk test. The non-parametric Kruskal–Wallis and Mann–Whitney *U*-tests were employed to make comparisons. We employed further post hoc testing using the Bonferroni correction method if associations were found. We conducted descriptive and associated analyses in R 4.2.2 (R Core Team, 2022). For all statistical analyses, significance was set at *p* < 0.05.

## 3 Results

### 3.1 Species

Over the five sampling surveys, we captured 519 eels. Using the identification key based on tail pigmentation (Réveillac et al., 2009), we positively identified 231 individuals (42%) across 3 species, namely *A. bengalensis* (*n* = 51), *A. marmorata* (*n* = 60) and *A. mossambica* (*n* = 120) (Figure S1). We captured all 519 eels during the high-flow surveys, with 113 caught in November, 403 in December 2021 and 3 elvers in March 2022. No eels were captured during the low-flow surveys in August and September 2022. Some individuals could not be identified to the species level as pigment patterns did not match the key.

### 3.2 Life stages and sizes

**Figure 2:**
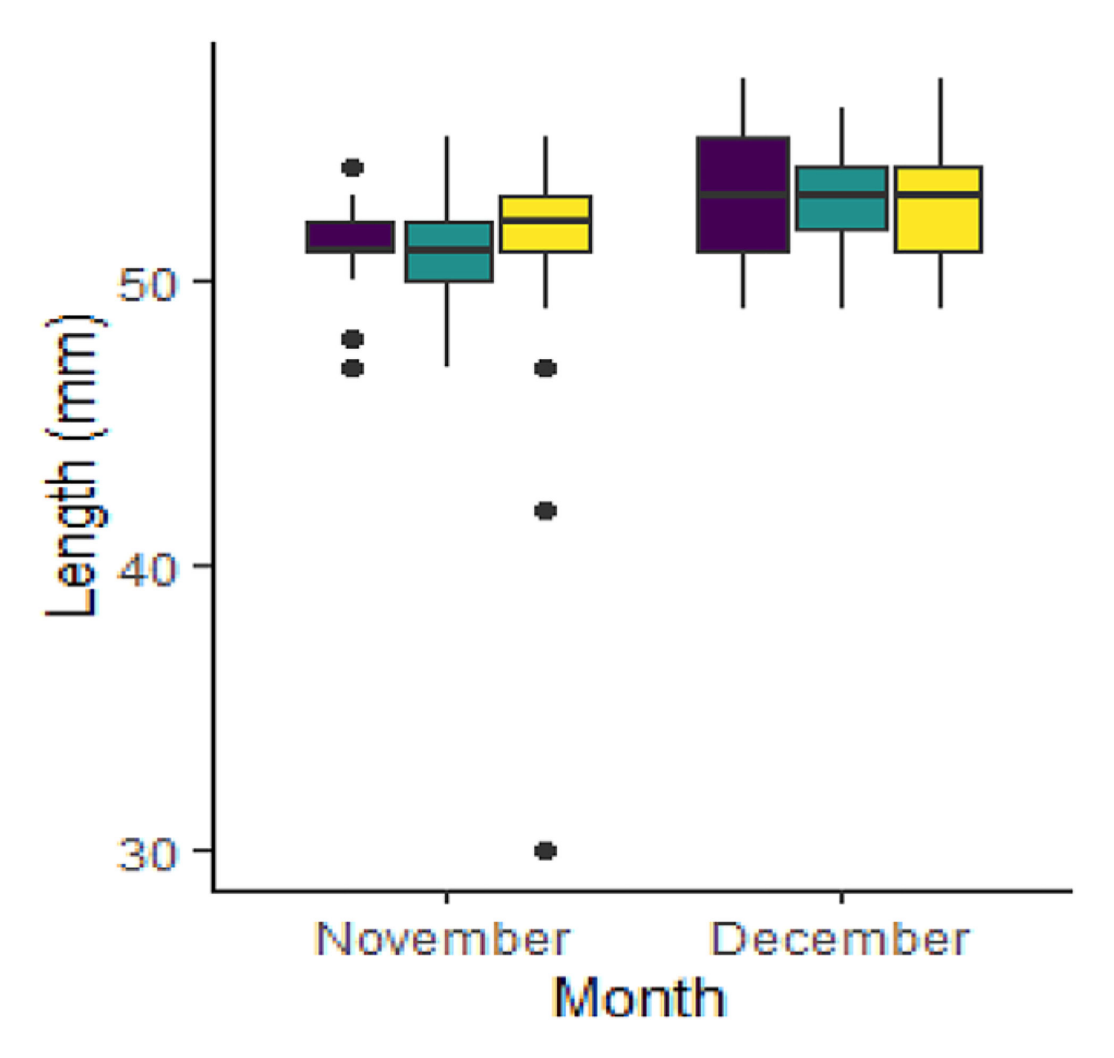
Whisker box plots detailing the total length of each *Anguilla* species, for each month, caught in the uThukela River, KwaZulu-Natal, South Africa, in November and December 2021 and March 2022. Coloured left to right for each period as *Anguilla bengalensis* (purple), *Anguilla marmorata* (green) and *Anguilla mossambica* (yellow).

**Figure 3:**
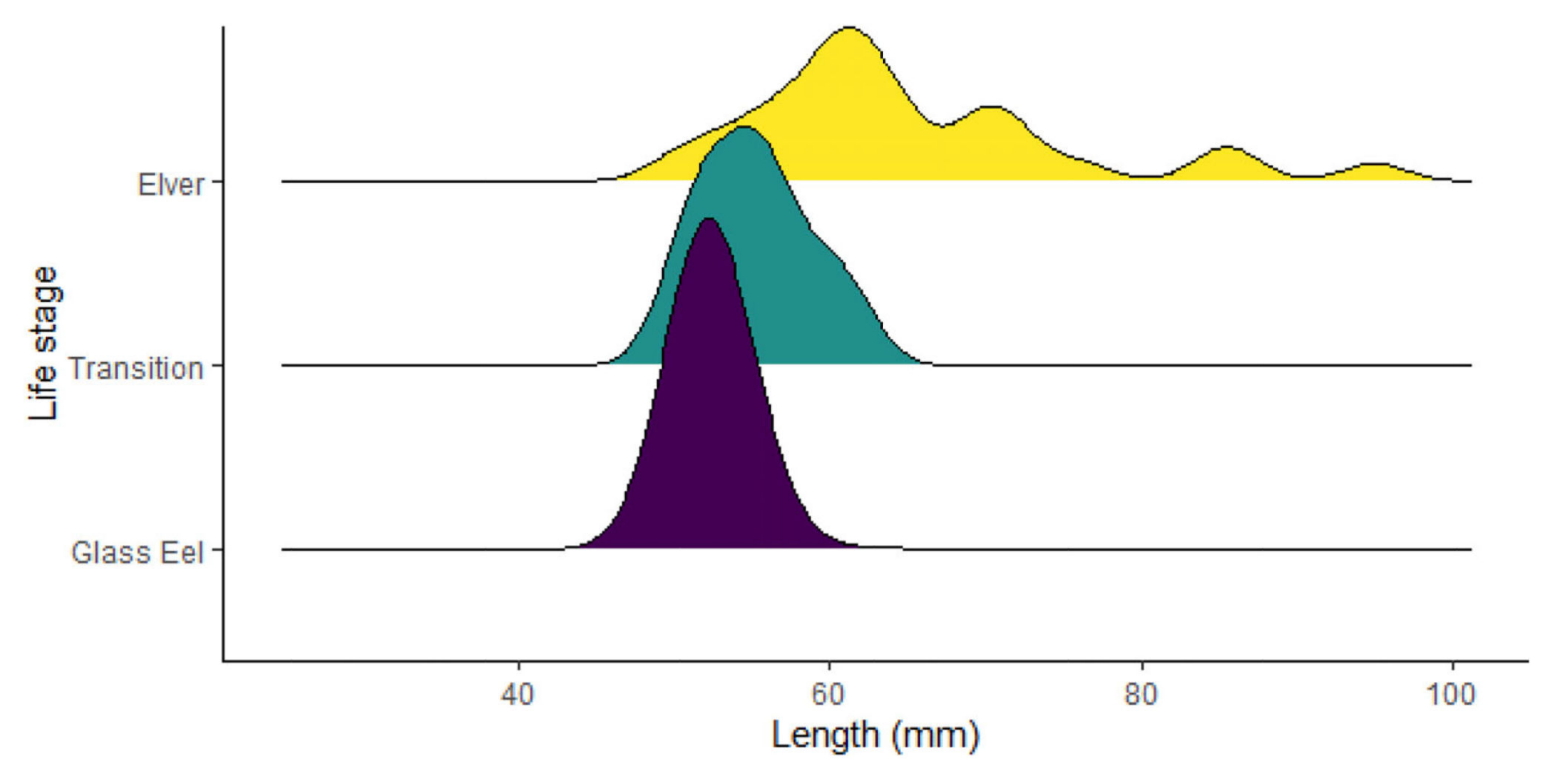
Density plot detailing the frequency (number of eels) of length (mm) for each life stage caught in the uThukela River, KwaZulu-Natal, South Africa, in the present study.

Of the total captured, 461 (88.8%) were glass eels, 36 (6.9%) were elvers and 22 (4.2%) were identified as being in transition between the glass eel and elver phase (Figure S1). Recruiting eel body lengths varied between species. These findings were similar to those for the *A. mossambica* and *A. marmorata* found in other studies, such as the Réunion study (Robinet, Lecomte-Finiger, et al., 2003; Table 1), with no comparable data for *A. bengalensis*. The lengths varied between recruiting stages, with lengths increasing from glass to transition to elver as expected. Eels caught in the high-flow surveys ranged in length from 33 to 95 mm, with a mean ± SD of 53.2 ± 4.83 mm. The mean (±SD) length of the eels for the respective identified species was as follows: *A. bengalensis* 52.4 ± 2.38 mm, *A. marmorata* 51.9 ± 2.04 mm and *A. mossambica* 52.2 ± 2.93 mm (Figure 2; Table 1). The body length linked to the eel’s life cycle stage (glass eel/elver/transition) indicated that glass eels ranged in length from 33 to 73 mm, transition-phase eels ranged from 47 to 61 mm and elvers ranged from 50 to 95 mm (Figures 3 and S2; Tables 1 and 2).

The length distribution (species and stages) was determined to be non-normal (Shapiro–Wilk test, *W* = 0.65714, *p* < 2.2 x 10^–16^). As distributions of lengths were non-normal, a non-parametric Kruskal–Wallis test was used to determine if there were significant differences between (a) the body lengths of the four groups and (b) the body lengths of the three stages. We found a significant difference indicating that body length differs among the four species groups (Kruskal–Wallis test, χ^2^ = 14.106, *df* = 3, *p* = 0.002764). Further post hoc testing was conducted using the Bonferroni correction to compare the lengths of the three identified species and the unidentified group. Results indicated that species’ body length did not differ significantly between each of the three identified species; however, it did differ significantly between *A. marmorata* and the unidentified individuals (Table 3).

There was a significant difference in body length among the three stages of eels (glass eels, elvers and transitional phase) (Kruskal–Wallis, χ^2^ = 78.823, *df* = 2, *p* < 2.2 x 10^−16^; Figure 3; Table 3). However, this test did not specifically identify which life stages differed from the other. To determine this, we conducted further post hoc testing using the Bonferroni correction. Results indicated that body length differed significantly between (a) elvers and glass eels and (b) elvers and transitional phase eels. However, the body length did not differ significantly between glass and transitional phase eels (Table 3).

### 3.3 Environmental variables

With regard to the environmental variables, the mean and ±SD values of all variables (pH, electrical conductivity, total dissolved solids, water temperature [°C], photoperiod and moon phase) are presented in Table 4. In terms of water quality parameters, we found that there was a significant difference between the high- and low-flow surveys for each variable (for all parameters, Mann–Whitney *p* < 0.001; Table 4). The pH, electrical conductivity, total dissolved solids and moon phase were lower under high-flow than low-flow conditions. In contrast, water temperature and photoperiod were higher under high-flow than low-flow conditions.

## 4 Discussion

Our study explored the recruitment of eels into the uThukela River and the associated variables (water flow, pH, electrical conductivity, total dissolved solids, water temperature, light quality and moon phase). The flooding event impacted data collection and presented higher-than-normal base low flows during the low-flow period (austral winter). Despite this, of the 519 eels we captured, we did not detect glass eels during the low-flow sampling, and even in March (austral autumn), the capture of glass eels had already decreased.

In a series of studies from the Réunion Island, 633 (Robinet, Guyet, et al., 2003; Robinet, Lecomte-Finiger, et al., 2003), 1978 (Robinet et al., 2007) and 4172 (Robinet et al., 2008) glass eels were caught during different studies using various methods and periods from 2001 to 2006. From October through February 2005 and 2006, Robinet et al. (2008) sampled Western Indian Ocean archipelago islands as far north as the Seychelles and included Madagascar. These studies indicate the variability in recruitment across the Western Indian Ocean over different seasons. The present study was one of the few to understand and establish the timing of recruitment of eels into the African mainland, specifically within their southern distribution limits.

Most eels in the present study were captured over November and December, supporting the recruitment of glass eels during these warm, high-flow months (austral spring–summer) in South Africa (Wasserman et al., 2011; Wasserman & Strydom, 2011). This concurred with findings from the Réunion and Mauritius islands (Robinet et al., 2007; Robinet, Lecomte-Finiger, et al., 2003). Robinet, Lecomte-Finiger, et al. (2003) found that most recruitment occurred between November and April, warm, high-flow months. In our study, during March 2022, the number of glass eels captured on the uThukela River was low (*n* = 3). This was comparable with the number captured (*n* = 10) by Robinet, Lecomte-Finiger, et al. (2003) in March 2001 in the Roches River on Réunion Island. However, in contrast, many more eels (*n* = 353) were captured in the Mat River on Réunion Island in March 2001 (Robinet, Guyet, et al., 2003). These studies differed slightly from a Kenyan study where glass eels were captured (*n* = 56) from July to October from the Sabathi and Ramisi rivers (Gitonga et al., 2022). These rivers are equatorial, and the timing of recruitment above the equatorial line is not fully understood. Our study sheds light on the southernmost distribution and recruitment of these species.

The 58% of unidentified species using tail pigmentation of recruiting eels in the present study was not surprising, as the relevance of the key by Réveillac et al. (2009) is not universally supported; for example, Robinet, Lecomte-Finiger, et al. (2003) reported difficulty using a key. Réveillac et al. (2009) noted that the key was useful as it could reliably distinguish *A. bengalensis, A. marmorata* and *A. mossambica* in the Southwestern Indian Ocean. However, in their 2003 study, researchers on Réunion Island found they could not separate *A. mossambica* and *A. bengalensis* based on tail pigmentation. Like Robinet, Lecomte-Finiger, et al. (2003), we found the key a challenge. The tail pigmentation of eels captured in our study site was not as discrete and species specific as depicted in the key of Réveillac et al. (2009). A sub-sample from the present study was used to find a more robust way to determine species type using genetic testing (Hanzen et al., 2020), and future research could consider this, although it is expensive.

The present study’s species composition was similar to that of the Madagascar catch from Robinet et al. (2008), which was dominated by *A. mossambica*. However, *A. bengalensis* was not caught in the Western Indian Ocean archipelago region (Robinet et al., 2008), unlike in the present study. The proportion of the other species, *A. marmorata* and *A. bengalensis*, observed in the present study is similar to the observed yellow eels in KwaZulu-Natal from 2015 to 2020 by Hanzen et al. (2022). *A. bicolor* was not found in our study, although it is usually confined to the lower reaches of rivers, and its detection in KwaZulu-Natal was low. Although its distribution in this region is considered extralimital, its abundance is also declining (Hanzen et al., 2022). Inland penetration may play an important role in the abundance and distribution of the different species, with *A. mossambica* able to scale steep gradient rivers and penetrate the furthest inland (Hanzen et al., 2022). The KwaZulu-Natal rivers have high gradients, and African eels with weaker climbing abilities are confined to the lower reaches and estuaries. Regionally, this is concerning for species, like *A. bicolor*, confined to lower reaches and facing increasing anthropogenic pressures (Wade et al., 2021). A Kenyan recruitment study found higher abundances of *A. bicolor*, supporting that there is a preferred distribution range for the four African eels (Gitonga et al., 2022).

The recruiting life stage of our study predominantly consisted of glass eels, followed by elvers and then transitional phase eels. The abundance of glass eels 8 km upstream of the uThukela Mouth opening in November and December suggests that the glass eels spend a relatively short period in the estuarine environment before moving upstream towards the freshwater environment, as found in other studies (Robinet, Guyet, et al., 2003; Lin et al., 2012). This relatively short period in the estuary was observed for eels recruiting into the Roches River on Réunion Island (Robinet, Lecomte-Finiger, et al., 2003). This may reflect the present study’s site in the upper estuary zone, away from the tidal zone. Here, the present study’s findings are in contrast to findings from Réunion Island, where the study site was much closer to the river mouth, with a predominance of glass eels and proportionally fewer numbers of transitioning eels and elvers (Robinet, Lecomte-Finiger, et al., 2003). Our study, therefore, would indicate that glass eels are sequentially metamorphosing as they move away from the river mouth, which is expected. However, this complexity becomes more apparent and unpredictable when associating the various stages of eel development with their respective aquatic habitats.

The lack of eels found in March in our study was contrary to the literature (Robinet et al., 2008; Robinet, Guyet, et al., 2003; Robinet, Lecomte-Finiger, et al., 2003). The extent of the flooding and extended periods of high flow may have negatively impacted the recruitment of glass eels during this period, and the extended flooding further disrupted the March 2022 survey. The uThukela River estuarine gradient is high, and the extent of the estuary is relatively small in high-flow conditions, especially during flooding events, compared with other river estuaries in the region (de Lecea & Cooper, 2016). This may have impacted the glass eels’ ability to use their selective tidal stream transportation, which would have been reduced during flooding to the extent experienced in the study (Harrison et al., 2014). If glass eels could not use selective tidal stream transportation in the uThukela River under high-flow conditions, they might have faced increased energy expenditure above usual limits.

No eels were found in the August and September 2022 (austral winter) low-flow study periods. It is difficult to comment conclusively on this, as this period followed the unusual flooding events, and these floods increased the expected low-flow baseflow for this period; however, water temperature and turbidity were in normal ranges for this period. Yellow eels are known to be less active during the winter in the uThukela River (Hanzen et al., 2021). Other studies show that eel recruitment is reduced from March onwards in the Western Indian Ocean. For example, Robinet, Lecomte-Finiger, et al. (2003) captured no eels in Réunion from May to October 2001. A further study concurred that the density of eels decreased after March (Robinet, Guyet, et al., 2003). We suggest future work and focus on eel recruitment into mainland Africa; our study highlights this need.

All measured environmental factors showed significant differences between the high- and low-flow surveys (pH, electrical conductivity, total dissolved solids, water temperature, photoperiod and moon phase). This was expected as the two flow states present very different seasonal conditions of the river and estuary. Warmer water, high flows and longer days (photoperiod) are characteristic of wet summers for the austral summer in the tropics and sub-tropical regions.

Water temperature generally positively affects eel recruitment (Jellyman & Lambert, 2003; Laffaille et al., 2007; Linton et al., 2007). In our study, recruitment occurred during the warm water temperatures that coincide with the high-flow period (austral summer), similar to other studies from the Western Indian Ocean region (Robinet et al., 2008; Robinet, Guyet, et al., 2003; Robinet, Lecomte-Finiger, et al., 2003). The photoperiod may differ between the present study site, as a sub-tropical site, and those in the Western Indian Ocean archipelago islands. The light intensity and moon phase were lower (23.3) during the high-flow survey than during the low-flow survey (72.2). Seasonality was determined as the main driver in this study, mainly because of high collinearity between season and moon phase (high-flow season sampling was conducted during new moon, and low-flow season sampling was conducted during full moon). The flooding event disrupted sampling and limited our data to test for seasonality. This seasonality was supported in a pilot study for the region (Africa, 2024).

Interestingly, the mean electrical conductivity values ranged between 287 (high-flow survey) and 358 (low-flow survey), possibly from the increased concentration of effluent during the low-flow period from industries upstream impacting the uThukela River (Wade et al., 2021). It is unclear what impact this may have on eel recruitment, especially during the low-flow period, when recruitment was shown to be low.

## 5 Conclusions

The timing and duration associated with the drift of leptocephalus larvae from their spawning area and the sequential penetration of glass eels into estuaries are still largely unknown and understudied, especially for the African eel species in the Western Indian Ocean. The distribution of these species and the timing at which they reach the continental rivers are still much debated. The present study contributes to this global discussion, showing further support for distribution patterns and migration pathways unique to the four African eels in the Western Indian Ocean. Our low identification of eels at the species level highlighted the need to improve the identification key for eels, especially for transitioning eels; this includes the use of genetics (Hanzen et al., 2022) when possible. The spatial gradient and occurrence of transitioning, as well as the elver eels captured, were found to be below the upstream limit of the uThukela Estuary, occurring concurrently with glass eels. This is an important area for further study, as eel stages are not fixed but rather reactive to exogenous influences.

Austral winter rainfall around the south of South Africa, where eels occur, which is not discussed in the present study, has very different climatic conditions from the uThukela River. The austral, equatorial and boreal summer rainfall may emphasise that larval drift is an important factor for recruitment into African rivers. Seasonality was the overriding factor in the present study; however, larvae drifting from spawning areas need attention, and patterns associated with this life stage of the Western Indian Ocean region’s eels are lacking. This highlights the importance of collaborative regional studies to better understand the Western Indian Ocean anguillid eels in Africa.

## Supplementary Material

Additional supporting information can be found online in the Supporting Information section at the end of this article.

Supporting Information

## Figures and Tables

**Table 1:** A comparison between eels captured in the uThukela River, South Africa (present study), and the Roches River, Réunion Island (Robinet, Lecomte-Finiger, et al., 2003).

Species	Length (mean ± SD) in the present study (mm)	Length (mean ± SD) in the Reunion study (mm)
*Anguilla bengalensis*	52.4 ± 2.38	
*Anguilla marmorata*	51.9 ± 2.04	54.5 ± 2.0
*Anguilla mossambica*	52.2 ± 2.92	52.4 ± 2.6

Abbreviation: SD, standard deviation.

**Table 2:** Numbers of each species and life-stage composition of eels caught for the duration of the present study.

Species	Glass eel	Transition	Elver	Total
*Anguilla bengalensis*	50	1		51
*Anguilla marmorata*	58	2		60
*Anguilla mossambica*	115	5		120
Unidentifiable	238	14	36	288
Total	461	22	36	519

**Table 3:** Summary of the Bonferroni post hoc test comparing eel species’ mean lengths and each life stage to one another in the present study.

Species comparisons	*Anguilla bengalensis*	*Anguilla marmorata*	*Anguilla mossambica*	
A. *marmorata*	1	-	-	
*A. mossambica*	1	0.8261	-	
*Anguilla* spp.	0.3977	0.0092*	0.1280	
**Life-stage comparisons**		**Elver**		**Glass eel**
Glass eel		<0.0001		-
Transition		<0.0001		0.024

* Kruskal-Wallis, chi-squared = 78.823, *df* = 2, *p* < 2.2 x 10^-16^.

**Table 4:** Summary of the mean (±SD) of environmental variables and the Mann–Whitney *U*-test results comparing the means of variables between high- and low flows in the present study.

	Mean ± SD			
Parameter	High flow	Low flow	*W*	*p*-Value
pH	7.4 ± 0.31	7.7 ± 0.55	2452	3.877E-06
Electrical conductivity (mS/cm)	287.8 ± 71.34	358.3 ± 53.94	2160	5.123E-08
Total dissolved solids (ppt)	146.4 ± 31.09	254.8 ± 37.98	0	<0.0001
Water temperature (°C)	26.1 ± 2.56	20.6 ± 2.80	7600	<0.0001
Photoperiod (h)	13:01:42 ± 0.10	11:30:26 ± 0.02	7680	<0.0001
Moon phase (% illumination)	23.5 ± 18.97	72.8 ± 13.22	128	<0.0001

*Note*: Only glass eels were detected in the high-flow period. Abbreviation: SD, standard deviation.

## Data Availability

The data belong to the University of KwaZulu-Natal and are available from the authors on reasonable request.
