# Supplementary material for: Life‐stage and environmental influences on the recruitment of African freshwater eels into the uThukela River, South Africa
Source: J Fish Biol. 2025 Sep 30;108(1):307–17. doi: 10.1111/jfb.70239 (PMC7618467; doi:10.1111/jfb.70239)
Supplement: Supplementary file 1 — Figure S1. An example of the image taken for each eel showing scale, date of capture, net of capture, effort number and individual number. This eel was identified as Anguilla mossambica as well as being in the transition phase between glass eel and elver, where it has some pigment development. Figure S2. An example of the difference between a glass eel (identified as Anguilla bengalensis) and a fully pigmented elver. [file JFB-108-307-s001.docx]

**
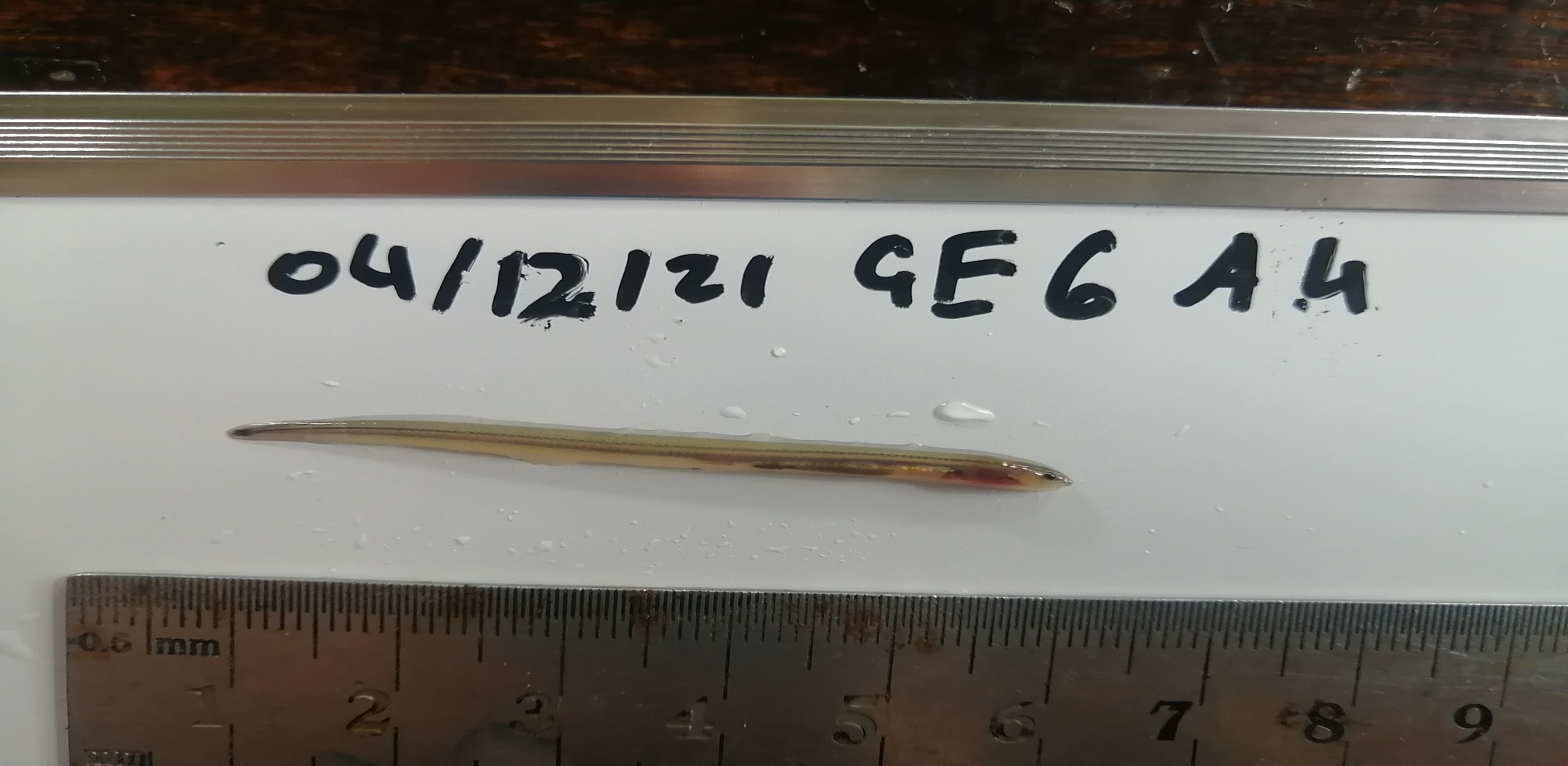
SUPPLEMENTARY INFORMATION**

**Supplementary information Fig. S1:** An example of the photograph taken for each eel; showing scale, date of capture, net of capture, effort number and individual number. This eel was identified *A. mossambica* as well as being in the transition phase between glass eel and elver, where it has some development of pigment.


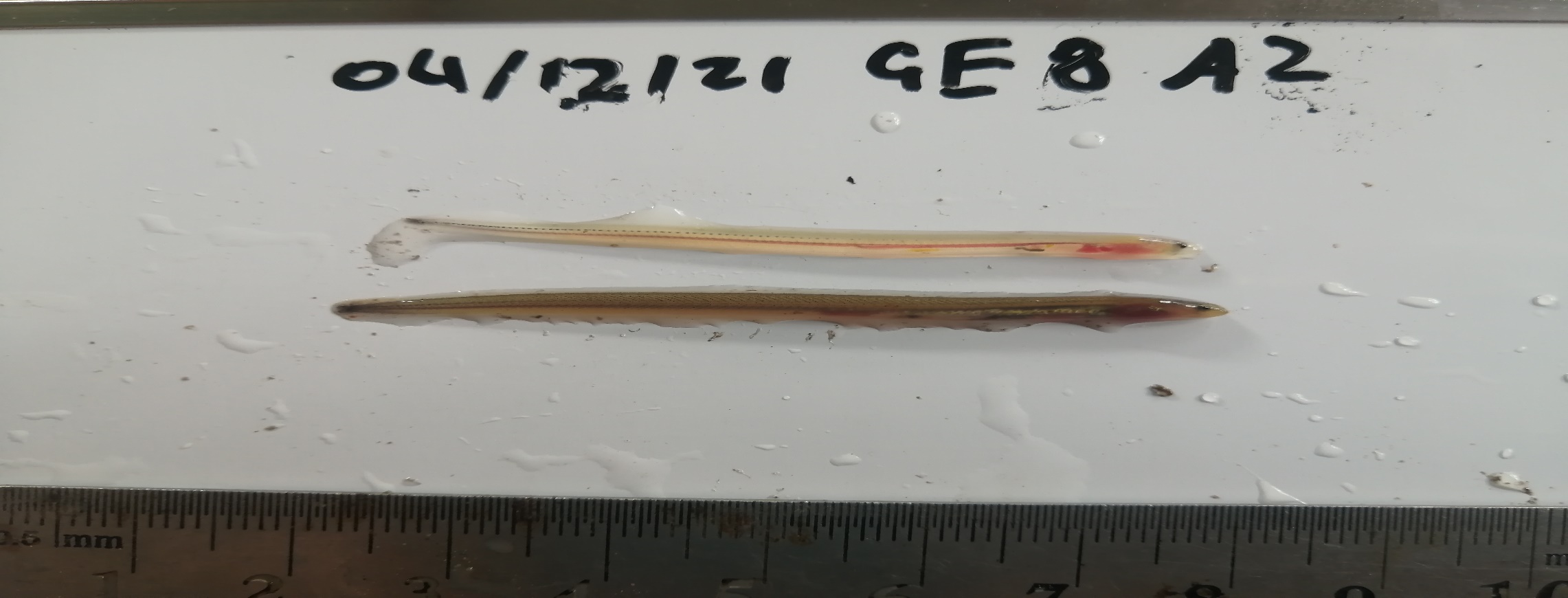


**Supplementary information Fig. S2:** An example of the difference between a glass eel (identified as *A. bengalensis*) and a fully pigmented elver.
